# Transitioning from the Emergency Department to a General Internist Outpatient Clinic for Paracentesis: A Qualitative

**DOI:** 10.21203/rs.3.rs-3793244/v1

**Published:** 2023-12-28

**Authors:** Jennifer Koh, Shadi Dowlatshahi, Barbara J Turner

**Affiliations:** Keck Medical Center of University of Southern California; Los Angeles General Medical Center; Keck Medical Center of University of Southern California

**Keywords:** paracentesis, care transition, qualitative research, implementation outpatient clinic, emergency department, safety net hospital

## Abstract

**Background:**

Repeated paracentesis for ascites can place significant demands on the emergency department (ED). A new general internist-led outpatient procedure clinic to alleviate this demand required ED staff and patients to accept this transition of care.

**Aim:**

This qualitative study evaluates barriers and facilitators to implementing the FLuid ASPiration (FLASP) clinic in a safety net hospital.

**Methods:**

The FLASP clinic opened during the COVID-19 pandemic in March 2021. From February to April 2022, semi-structured interviews were conducted with: 10 ED physicians and nurses; 5 FLASP clinic patients; and 4 patients receiving paracentesis in the ED. Interviews were recorded, transcribed, and analyzed using a Grounded Theory approach for themes categorized by Theory of Planned Behavior (TPB) domains including: attitudes/knowledge; social norms; and logistics.

**Results:**

Thematic analysis found that ED staff appreciated reduced demand for paracentesis, but barriers included: lack of knowledge; concerns about unstable patients and patient expectations (norms); and scheduling logistics. FLASP clinic patients had only favorable themes: belief in clinic safety; positive relationship with staff; and clinic efficiency. Patients using the ED for paracentesis expressed only concerns: possible need for testing or hospitalization; care usually in the ED; and unclear clinic scheduling.

**Conclusion:**

This study reveals challenges to transitioning sites of care for paracentesis including the need for greater ED staff education and standardizing methods to triage patients to appropriate site of care. Greater support and education of ED patients about the benefits of an outpatient procedure clinic may also reduce ED burden for paracentesis.

## Introduction

When ascites becomes refractory to medical measures, it can be managed by repeated paracentesis but, optimally, transjugular intrahepatic portosystemic shunt (TIPS) or liver transplantation^1^ can avert the need for paracentesis. However, these interventions are less accessible to low income populations in the U.S.^2^ Consequently, uninsured or underinsured patients with refractory ascites continue to place significant demand on the emergency department (ED) and inpatient hospital setting to perform paracentesis.^3^ Although outpatient clinics have been increasingly established to perform paracentesis^4–7^, these services have frequently been delivered by interventional radiologists^8^ and may not be available in safety net institutions caring for low-income populations.

At one of the largest safety net hospitals in the nation, the imperative to reduce the burden on the ED during the COVID-19 pandemic prompted a hospitalist to establish the FLuid ASPiration (FLASP) clinic to perform outpatient ultrasound-guided paracentesis. Implementation of the clinic involved expansion from part-time to full-time weekdays hours to increase accessibility and adding a full-time nurse-practitioner and rotating internal medicine resident physicians to the clinic personnel.

However, implementation of the FLASP clinic required changes in behavior and medical care norms of referring ED providers and patients. Scant evidence is available from the vantage point of these key stakeholders to inform a successful launch of such a clinic. To address this gap, we conducted semi-structured key informant interviews with patients and providers regarding barriers and facilitators to implementing in outpatient clinic according to Ajzen’s Theory of Planned Behavior, a widely used conceptual model to assess behavioral change.^9^ This qualitative inquiry examined themes regarding attitudes/knowledge, social norms, and logistical issues (perceived behavioral control). These perspectives offer valuable insights to inform similar efforts on the implementation of outpatient paracentesis clinics as a potentially valuable alternative to urgent care settings.

## Methods

### Overview of FLASP Clinic Implementation

Demands on a safety net institution’s ED for patient care during the COVID-19 pandemic prompted a hospitalist physician to propose implementation of a dedicated outpatient clinic to alleviate the need for routine paracentesis in the ED. To obtain approval for this new service, the hospitalist physician met with key stakeholders including the institution’s chief medical officer and several leaders of ED. Hepatologists at this safety net institution did not perform any paracenteses, so the outpatient clinic would not compete. After approval of initial staffing and space, the hospitalist director met with ED leadership as well as some ED clinicians and staff to introduce the clinic and agree upon referral procedures.

The new FLASP clinic was launched in March 2021. To meet increasing patient demand for care in FLASP clinic, hours of operation expanded to all weekdays and staffing increased from the physician director and a nurse to include a nurse practitioner, part-time internist faculty, and rotating house staff. Clinicians who joined the FLASP clinic were instructed to perform the paracentesis according to American Association for the Study of Liver Diseases guidelines ^10,11^ and the physician director observed each one performing at least five procedures. ED staff could use the EMR to schedule patients for an appointment in the FLASP clinic and patients received phone calls reminding them about appointments.

### Quantitative Interviews

Critical to evaluating the quality and safety of all procedures related to the FLASP clinic was learning directly from clinicians and staff of the ED who referred patients as well as from patients received care in FLASP or continued to receive care in the ED. Key informant interviews to garner these perspectives regarding implementation of the FLASP clinic were conducted from February 1st, 2022, through April 30th, 2022. We aimed to learn from diverse patient perspectives, so the research team sought to engage a sample of patients who received paracentesis in the FLASP clinic and a sample of patients who continued to utilize the ED. As the main source of patient referrals to the FLASP clinic, the team also aimed to conduct key informant interviews with a sample of nurses and physicians staffing the ED. Persons not delivering care in the FLASP clinic or ED conducted interviews in-person or by telephone. All interviews were recorded via audio and lasted about 15 minutes. Interviewers assured the subjects of their anonymity with names deleted after the recording. The University of Southern California Institutional Review Board approved the project (UP-20-01435).

### Subject Eligibility and Recruitment

Eligible patient subjects were identified from the electronic medical record as only utilizing the FLASP clinic after launch or continuing to utilize the ED for paracentesis. The timeframe for assessment of patient use of FLASP clinic or ED care was from November 1, 2021, to April 30, 2022, with semi-structured key informant interviews conducted from February through April 2022. *A priori*, we aimed to interview five key informant subjects from each of the following groups: FLASP clinic patients, ED patients, ED physicians, and ED nurses.

For the first group, FLASP clinic patients were approached by a study team member (not the clinic director) on different clinic days for the brief interview either in English or Spanish using hospital standardized interpreting services. The team member described the study and reviewed the consent form. After signed consent, the 15 to 20 minute interview was conducted in clinic and recorded with consent.

The second group of ED patients were contacted by telephone and, after a description of the study, asked to consent verbally. The telephone interview was also recorded. A unique challenge to recruiting ED patients was that they were often lacked reliable contact information or were unhoused. Therefore, four interviews were completed with ED patients but these patients all cited similar barriers.

The last two groups consisted of five ED nurses and five ED physicians. These participants were approached in the ED by a study member for the interview and, after a description of the aims of the interview, asked to sign a consent. The 15 to 20 minute interview was conducted in person and recorded with permission. None of patient or staff subjects refused after being invited to participate. All interviews were transcribed (and translated as needed) to text for analysis by the study team. No personal details were obtained from participants. For participation, all patients were provided with a $25 gift card.

### Data Collection

Interview guides developed by the research team featured focused and open-ended questions about barriers and facilitators to attending the FLASP clinic and preferences regarding ED care according to the TPB (available upon request). The semi-structured patient interview guide addressed the following aspects of care: knowledge/awareness about the FLASP clinic; personal experiences with providers in the clinic or the ED; social norms and experiences with safety at the FLASP clinic and expectations about urgent care; and logistic issues with accessing FLASP clinic. The guide for provider interviews focused on knowledge/awareness about the clinic as well as barriers and concerns with referring patients to the clinic and informational gaps. Ajzen’s Theory of Planned Behavior (TPB) was employed as a framework for analysis because emergent themes addressed key domains of this well accepted behavioral mode.^9,12^

### Data Analysis

The study adopted the Grounded Theory approach for qualitative research.^13^ We conducted a theoretical thematic analysis to identify patterns in semantic content of the transcribed raw data that could be codified into themes.^14^ The process was both inductive and deductive. Following recommendations by King^15^, this analysis was performed using a recursive process that was applied separately to the patient interviews and ED provider (physician and nurse) interviews. Three independent researchers (BJT, KH, SD) repeatedly read the transcripts and took notes regarding potential ways to code the data. These team members then reviewed their ideas for coding together and discussed agreements and differences. A consensus was reached regarding an initial set of codes for coding themes related to the Ajzen’s Theory of Planned Behavior (TPB), including 1) attitudes/knowledge and experiences with the FLASP clinic or ED care; 2) social norms regarding appropriate site for paracentesis; and 3) logistical factors that prevented or facilitated scheduling and/or attendance to the FLASP clinic or the ED (perceived behavioral control).^9^

## Results

Semi-structured, key informant interviews were completed with 5 FLASP clinic patients, 4 ED patients, 5 ED nurses, and 5 ED physicians to identify facilitators and barriers to patient utilization of the FLASP clinic. Themes from our qualitative analysis of interviews were categorized by domains of the Theory of Planned Behavior and summarized in Tables 1 (provider themes) and Table 2 (patient themes). Both tables reveal that knowledge and attitudes expressed by patients and ED clinicians/staff supported the value of the new FLASP clinic for its convenience, safety, and longitudinal provider-patient relationships. The ED interviewees greatly appreciated an alternative for paracentesis. Yet interviews also identified barriers to address concerning knowledge and attitudes of patients who desired immediate testing and evaluation in the ED and were unaware of the new clinic. Additionally, ED clinicians were unclear about appropriate triage to the FLASP clinic, and how to manage patients who valued aspects of ED care. Tables 1 and 2 reveal that lack of comments endorsing the FLASP clinic as a standard of care (social norm) even though attitudes of providers and patients indicated that it was becoming well accepted. The following sections offer specific quotes from patients and ED providers in regard to each theme.

### FLASP and ED Patient Interview Themes

#### Attitudes/Knowledge

Among the FLASP clinic patients interviewed, a common theme addressed the positive emotional support from the clinic staff as a facilitator to receiving this care.

No. I just want to say that the service is worth it, and they treat people as human beings, and they treated me like if I was a family member.

And they really talk to you, and they see how you’re doing and how you feel, and I think those are great points.

It’s better here [in the clinic], because I already know the people that are attending me.

Another theme related to the satisfaction with FLASP clinic care in achieving the goal of sustainable relief of ascites.

“I had [a paracentesis] once, and this experience is better. I get [more fluid] pulled out and it’s more - it’s more durable. It wasn’t with a bag, it’s with the bottles and I can see what’s going on.

Among the ED patients, the most prevalent attitudinal theme was the perception that more is done in the ED, but others noted that they were not aware of the FLASP clinic as an option.

Yes, I would prefer to go to the ED because they do a lot more tests on me than the clinic and I always get admitted to the hospital.

“The problem is that my doctor’s office makes me go to the ED [for further tests], and then they don’t give me an appointment [to the paracentesis clinic]”.

### Social Norms

For patients, the most common social norm was that their providers typically referred patients to the ED, or the ED was the usual site for this care.

”They usually send me to the ED from the [medical] clinic [to get my paracentesis], and so I’m not familiar with [FLASP], but now I am aware of the clinic. “

I wouldn’t be able to tell [the difference] because I can’t compare. I have always had it done in the ED, so I don’t know anything else.

#### Logistical Issues (Perceived Behavioral Control)

A common theme for FLASP clinic patients was the convenience of this care.

“It seems like everything that they do, they do it correctly and they do it fast, to get you in and out.”

I like to come here because I am a well-attended, and they see me fast.

Similarly, a theme that emerged from FLASP clinic patients as potential facilitator was the perception that it took more amount time for ED care.

“It took 18 hours to get the procedure done in the ED, so I would prefer to get in the clinic where it is safer.”

“In the ED, if I go to ED at 10:40 AM and then I have to wait there sometimes to the next day to have the fluid taken out. They take a very long time compared to the clinic. I can just go straight to have the procedure done.”

For both FLASP and ED patients, dominant themes related to barriers to FLASP clinic care were related to scheduling care and needing more intensive or urgent care.

Sometimes I don’t remember my appointment and I don’t know when it is.

The last time I was admitted [into the hospital], I was supposed to get an appointment of when to come to the clinic, but I have not received anything as of yet. There is a communication problem of knowing when the appointments are.

Yes, because I get an appointment and then I get sick and I can’t remember when my appointment is, so I come to the ED.

Yes, I would prefer to go to the ED because they do a lot more tests on me than the clinic and I always get admitted to the hospital.

### Provider Interview Themes

#### Attitude/Knowledge

Among the ED physicians and nurses, a dominant theme was the belief that the FLASP clinic alleviated burdens for care in the ED.

I feel like it’s a little bit more beneficial for us too, because we get more rooms for non-paracentesis patients, which again, procedures could take hours or so and it’ll give us a bed sooner than later.” “The story I get from the ED attendings is that we used to do a ton of paracentesis in the ED […] And then this procedure clinic opened up for paracenteses and a lot of our patients go there now.

Another emergent theme was satisfaction with the care that was provided in the FLASP clinic and hearing from patients about their positive experiences.

I’ve seen it and I’ve been there [to the clinic], and I think it looks fantastic. It’s like wow – I’m very happy patients can go there, easy access. They can go in and out [of the clinic], instead of coming through the ED, waiting in line.

“And then this procedure clinic opened up for paracenteses and a lot of our patients go there now, and so we do a lot less of them in the ED, and it seems to work well for the patients.”

Among the ED physicians, a common concern emerged regarding referral to the FLASP clinic and the need to assess whether the patient might require hospitalization.

“I want to make sure that the underlying reason for why they need this procedure isn’t one that needs to be admitted for. And sometimes I feel like I need this procedure to help me determine that.”

“I guess just with having certain parameters in place, just to make sure that they have maybe previous lab results or no other underlying issues prior to going to the clinic, such as elevated ammonia levels or just anything that could make them come back to the ED once they’re there to begin with.”**Social Norms**The ED providers commonly described difficulties with changing established practices and expectations for patients that arrive in the ED.

“It was challenging to get [the patients] to reframe that what they were experiencing was not an emergency and that they could in fact get [the paracentesis] done much faster at an outpatient clinic.”

“The patients who are exceedingly uncomfortable physically from, let’s say, their volume overload in their belly, who are expecting the immediate fix of the paracentesis right there in the E.D. - it’s been difficult to convince them to wait another day to go to the clinic.”

Initially at first when this clinic first started …we had some patients who were a little bit upset that they couldn’t do the paracentesis the same time here in the emergency room. Essentially, they’re wanting to come to the emergency room [to get] it done, and they were a little bit upset that we had to send them off to an outpatient thing, where they [were] essentially waiting for a procedure.

#### Logistical Issues (Perceived Behavioral Control)

ED physicians and nurses were generally unaware that FLASP clinic had been established or had questions about how to rapidly refer and schedule appropriate patients.

“I haven’t referred [anyone to the clinic] because I didn’t know about it […] Well, I guess, just a lack of information probably.” “No. Just, I mean, maybe a list of what [type of patient] can be referred and where the location [of the clinic] is. And maybe a little map if we handed out to our patients that are coming in, that would be great.”

And then, I think barriers are probably more so in scheduling conflicts with how many patients need procedures and the availability of the procedure clinic itself.

I mean, the only challenge that I would be aware of is that - Can we refer them straight from, if they come in through the ER? It’s my understanding that we have to check everybody in the ER. So that would be nice to have a protocol in place to refer them [to the clinic].

## Discussion

Implementation of novel health care delivery settings requires not only complex training of providers and staff^16^ but also behavioral changes by patients and referring providers to understand, accept, and utilize an alternative to the current pattern of care.^17^ This qualitative study used Ajzen’s Theory of Planned Behavior as a conceptual model^9^ to categorize facilitators and barriers to transitioning care for patients with refractory ascites from the ED to the FLASP procedural clinic. Although management of refractory ascites with repeated paracentesis has been reported to be less cost effective than the TIPS procedure ^18,19^, low-income populations such as those served by the safety net institution in this study confront significant barriers to accessing TIPS. Thus, paracentesis offers an important management option until, ideally, patients can receive either TIPS or liver transplant.

Patients’ experience with an outpatient clinic for paracentesis has been described in few studies. One study reported high patient satisfaction in a nurse-led paracentesis clinic.^20^ Yet to date, we are unaware of any qualitative studies of factors influencing adoption by patient or provider stakeholders for an outpatient procedural clinic such as FLASP. In this qualitative study, we aimed to identify actionable themes that, if addressed or promoted, could facilitate transition from ED to a procedure clinic. The FLASP clinic was launched expeditiously to alleviate unnecessary patient care in a safety net institution’s ED during the COVID-19 pandemic and might have missed important implementation steps.

**Figure 1:**
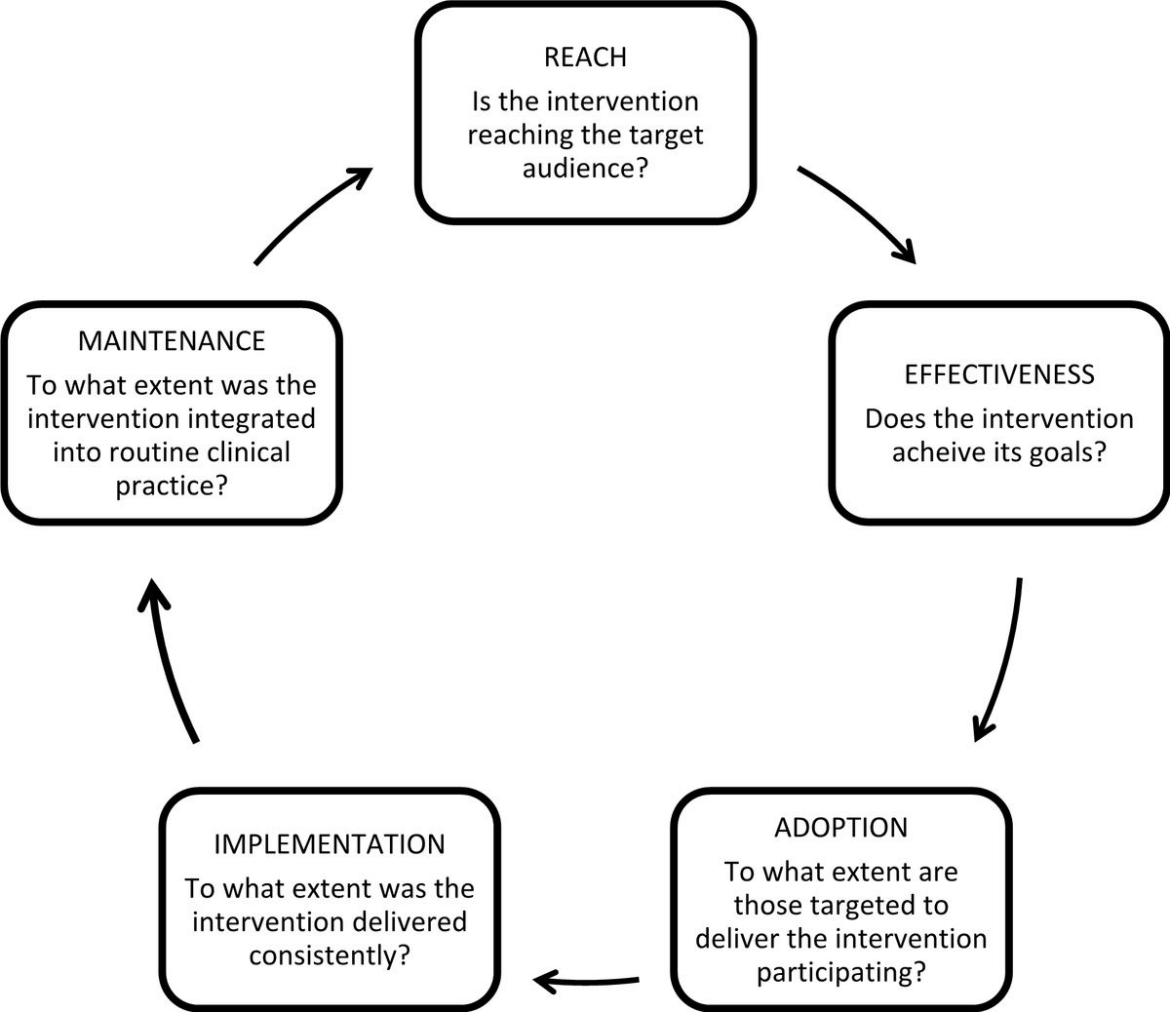
RE-AIM Quest* Domains For Implementation Research * Adapted from Forman et al^22^

According to the Agency for Healthcare Research and Quality (AHRQ), strategies for improving ambulatory care should focus on: access to care and information; communication with patients; coordination of care; customer service; and health promotion/education.^21^ This AHRQ plan of patient-oriented strategies emphasized on using easy and inexpensive approaches. However, analysis of implementation of a new service requires a more comprehensive model such as RE-AIM Quest, developed by Forman and colleagues ^22^ for qualitative assessment of Glasgow’s RE-AIM implementation framework.^23^ Fig. 1 displays five domains of RE-AIM Quest and associated questions. To address Reach and Adoption by patients, ED clinicians and staff were provided information about the clinic and trained to use the EMR for scheduling. In addition, ED staff members were sent email messages about the FLASP clinic but many did not receive in person education. The clinic was open every weekday to meet patient needs.

Gaps identified by the interviews revealed that patients needed low literacy information about the clinic explaining the value of this efficient, safe service as well as available testing services and rapid hospitalization if needed. Phone call appointment reminders were inadequate to overcome challenges with keeping appointments by historically marginalized patients.^24^ The small FLASP clinic team would have been well served by adding a care navigator or promotora to facilitate care. Care navigators have been endorsed as a valuable addition to the care team for advanced liver disease given the complexity of managing complications such as ascites and cognitive impairment.^25^ Studies of navigator care for patients with other complex conditions support their role in insuring receipt of needed care.^26,27^ Navigators for FLASP clinic patients could have addressed three domains of the Theory of Planned Behavior by increasing knowledge, reinforcing the social norm of outpatient paracentesis, and addressing logistical barriers to care. Sadly, limited reimbursement for these services has prevented integrating care navigators into many care setings.^27^

In terms of Reaching ED staff to facilitate implementation of the FLASP clinic (Fig. 1), interviews with ED nurses and physicians identified the need for more intensive efforts to establish clearer standards and logistics for referral. Although the American College of Emergency Physicians (ACEP) recommends treatment of all patients who present to the ED,^28^ FLASP clinicians and the ED team have collaborated on stablish standards for triage of stable patients to the FLASP clinic along with efficient referral procedures to insure timely care. ED clinicians will continue to manage less stable patients as well as those who prefer testing and evaluate in the ED.^29^ Indeed, a recent systematic review found that advanced triage protocols in the ED reduced length of stay without compromising safety or patient satisfaction.^30^

Regarding Implementation, (Fig. 1) the FLASP clinic leaders had to ensure availability of next day appointments for patients if needed. Delays in performing this procedure in hospitalized patients has been associated with longer lengths of stay and mortality.^31^ Delays are even more common for disadvantaged patients needing paracentesis.^32^ Staffing and clinic hours expanded to insure that all patients could be rapidly accommodated. Implementation also involved rigorous training of clinicians staffing the clinic to meet a high standard.

Effectiveness of care (Fig. 1) was endorsed by patients’ comments about safety, timeliness, and personalized care from FLASP clinicians. Longitudinal care from FLASP providers added value by educating patients about lifestyle changes to reduce the need for paracentesis and collaboration with the hepatology team for the TIPS procedure. Similarly, ED providers endorsed Effectiveness of timely, safe, and convenient care provided by FLASP clinic and appreciated it as an alternative for paracenteses. In a quantitative analysis, we reported that the FLASP clinic was associated with reduced demand for paracentesis in the ED.^16^ Lastly, Maintenance of the FLASP clinic (Fig. 1) was not specifically addressed by this qualitative study but patients and ED providers expressed satisfaction with the FLASP clinic, supporting the value of continuing this new service.

This qualitative study has several limitations. First, themes emerging in this qualitative study may not be generalizable to other health care settings. This study is most relevant to other safety net institutions nationally that provide care to uninsured and underinsured persons. Institutions such as ours rely heavily on Medicaid for funding along with supplemental financial support through Medicaid’s disproportionate share hospital (DSH) payment program.^33^ Clinicians at safety net institutions are often salaried without payment for providing specific services in contrast to for-profit or non-for-profit health care settings that bill CMS and other payers for health care services.^34^ In the latter case, hepatologists who perform outpatient paracenteses might take exception to a new clinic. On the other hand, the vast network of safety net institutions across the nation are also similar to ours in seeking innovative approaches to reduce ED overcrowding and patients leaving without being seen.^35^ To replicate this study, similar buy in from administrators would be required to commit staff and space and they would also need to endorse reducing demand on ED as a desirable goal.

A second limitation relates to our small sample size of interviewees. From the outset, we intended to interview 10 ED clinicians and nurses and 10 total patients from the ED and the clinic but only reached four patients receiving ongoing care in the ED. ED patients served by this safety net institution often have unstable housing or limited modes of communication. Yet our qualitative analysis revealed that these patients expressed similar reservations about attending the FLASP clinic. Third, the challenge in appropriately triaging patients with decompensated cirrhosis to receive outpatient versus inpatient care appears to be common.^36,37^

In conclusion, widespread adoption of accessible, safe alternatives to ED care for patients with refractory ascites has special value for safety net institutions such as ours serving the growing population of low-income patients with advanced liver disease.^38,39^ This study complements other qualitative studies of implementing ultrasound-guided thoracentesis for low income populations^40^ and supports the potential value of an implementation toolkit^41^ informed by themes elucidated in this study. Long-term sustainability may depend on expanding the array of procedures offered in the clinic to ensure that it continues to be highly utilized and alleviates demand by stable patients for urgent care services. This new model of care would also benefit from a formal cost-effectiveness analysis to inform dissemination to other institutions.

## Figures and Tables

**Table 1:** Patient Barriers and Facilitators to Adoption of the FLASP* Clinic for Paracentesis in a Safety Net Institution Categorized by the Theory of Planned Behavior ^9^

Theory of Planned Behavior Domain	Patient Facilitator Theme	Patient Barrier Theme

Attitudes/knowledge and experiences with FLASP or emergency department (ED) care	• Positive longitudinal relationships and support from FLASP staff	• Appreciate ED performing more tests for evaluation during visit

	• Satisfaction with FLASP care in sustainable relief of ascites	• Not aware of FLASP clinic option

	• Personalized attention	
	• Belief that FLASP is safer	

Social norms about appropriate site for paracentesis		• Doctors’ office only refers to the ED
		• Belief that norm is ED

Logistical factors influencing attendance to FLASP or ED†	• Quick service • ED much more time consuming than FLASP	• Forget FLASP appointment • Poor communication about appointments
		• Usually admitted to the hospital from ED so more appropriate

* FLASP FLuid ASPiration Clinic

**Table 2:** Emergency Department (ED) Provider Barriers and Facilitators to Adoption of the FLASP* Clinic for Paracentesis in a Safety Net Institution Categorized by the Theory of Planned Behavior^9^

Theory of Planned Behavior Domain	Provider Facilitator Theme	Provider Barrier Theme

Attitudes/knowledge and experiences with FLASP or ED care	• Value of having more ED rooms to care for other patients	• Need to assess the patient for a condition requiring hospitalization

	• Appreciate less demand to perform paracentesis	• Concerns about having conditions that would send them back to the ED

	• Excellent accessible care at FLASP clinic	
	• Belief that patients’ needs are met	

Social norms about appropriate site for paracentesis		• Their patients believe ED is appropriate for site for ascites care
		• Their patients come to ED wanting immediate relief for discomfort
		• Their patients decline referral to FLASP

Logistical factors influencing attendance to FLASP or ED	• Better protocols for referring appropriate patients	• Inadequate information about referral process
		• Better information about appropriate referral
		• Scheduling conflicts

* FLASP FLuid ASPiration Clinic
